# Tuning Shear Thinning Factors of 3D Bio-Printable Hydrogels Using Short Fiber

**DOI:** 10.3390/ma16020572

**Published:** 2023-01-06

**Authors:** Slesha Tuladhar, Scott Clark, Ahasan Habib

**Affiliations:** Department of Sustainable Product Design and Architecture, Keene State College, Keene, NH 03435, USA

**Keywords:** 3D bioprinting, shear thinning, rheology, shape fidelity, large-scale scaffold

## Abstract

Among various available 3D bioprinting techniques, extrusion-based three-dimensional (3D) bioprinting allows the deposition of cell-laden bioink, ensuring predefined scaffold architecture that may offer living tissue regeneration. With a combination of unique characteristics such as biocompatibility, less cell toxicity, and high water content, natural hydrogels are a great candidate for bioink formulation for the extrusion-based 3D bioprinting process. However, due to its low mechanical integrity, hydrogel faces a common challenge in maintaining structural integrity. To tackle this challenge, the rheological properties, specifically the shear thinning behavior (reduction of viscosity with increasing the applied load/shear rate on hydrogels) of a set of hybrid hydrogels composed of cellulose-derived nanofiber (TEMPO-mediated nano-fibrillated cellulose, TO-NFC), carboxymethyl cellulose (CMC), and commonly used alginate, were explored. A total of 46 compositions were prepared using higher (0.5% and 1.0%) and lower percentages (0.005% and 0.01%) of TO-NFC, 1–4% of CMC, and 1–4% of alginate to analyze the shear thinning factors such as the values of *n* and *K*, which were determined for each composition from the flow diagram and co-related with the 3D printability. The ability to tune shear thinning factors with various ratios of a nanofiber can help achieve a 3D bio-printed scaffold with defined scaffold architecture.

## 1. Introduction

A new technology commonly known as three-dimensional (3D) bioprinting uses scaffolding and carefully regulated cell and biomaterial distribution to replicate biological tissues [1,2]. The bio-fabrication process is heavily researched for regenerative medicine research for various application areas, including tissue engineering, transplantation and clinics, pharmaceutics, high-throughput screening, and cancer research [3,4,5,6,7]. This is because of its spatial and temporal deposition capability through the fabrication parameters. Out of seven Additive Manufacturing (AM) techniques defined by F42 the committee of American Society for Testing and Materials (ASTM) [8], Material Extrusion [9,10,11,12], Material Jetting [13,14,15,16,17], and Vat Polymerization [18,19,20,21] have been mostly used for 3D bioprinting processes. Following a vectorized toolpath, a variety of materials can be printed parallel to the XY plane, and the z-axis movement ensures the progressive 3D build height of the scaffold construct. The viscosity and density of the 3D bio-printable biomaterials encapsulating living cells, frequently referred to as bioink, must be appropriate for the printing technology. It must also maintain its shape for a predetermined amount of time and exhibit biocompatibility through good cell viability both before and after printing. Thus, three characteristics such as printability, shape integrity, and biocompatibility are necessary for the bioink to work properly [12]. Printability, shape integrity, and biocompatibility can be defined and measured with multiple characteristics such as material-wise: viscosity, pH, functional group, microstructure, and process and parameter-wise: printing pressure, speed, nozzle diameter, print distance, and temperature [22].

Because it is simple to prepare and has regulated rheological behavior, naturally derived sodium alginate is one of the hydrogel materials that is frequently utilized in extrusion bioprinting [23]. Even though higher weight percentage and higher molecular weight can increase the viscosity of materials, higher viscosity may jam the dispensing nozzle, requiring additional force to clear it, distorting the print and lowering cell viability [6]. Due to low modulus, achieving well-defined 3D shape, size, and dimensional integrity is challenging after printing. The deposited filament must possess sufficient mechanical strength to support the ensuing layers [3]. Making large-scale scaffolds also requires maintaining the shape accuracy of the scaffold, which requires little dispersion and filament sagging after printing [4]. Carboxymethyl cellulose (CMC) is a polysaccharide and cellulose derivative. It is used to change viscosity, has a large molecular weight, and is soluble in water [24]. Additionally, the binding of the CMC’s matrix protein aids in cell adhesion and movement [25]. To obtain improved physical and mechanical qualities, several researchers have mixed CMC with alginate. To enhance the mechanical and biological (for example, cell development) capabilities of the base hydrogel material, nano-scale reinforcements such as polylactic acid (PLA) nanofibers and nano-fibrillated cellulose (NFC) have been studied [26,27]. However, because of the unpredictable nature of the pressure needed and the non-uniformity of network entanglements in the pure NFC gel, it is extremely challenging to 3D print them [28]. To improve uniformity, dispersibility, homogeneity, and printability (Tempo-NFC), the surface of the NFC-based gel is altered by oxidation using 2,2,6,6 tetrame-thyl-1-piperidinyloxy (TEMPO) to add negatively charged carboxylate ions, termed as TO-NFC.

Shear stress is inevitable in any extrusion process and must be considered when selecting 3D bio-printable materials. Various printing process parameters such as nozzle diameter, printing pressure, and material viscosity influence the level of shear stress [29]. This factor even becomes vital if someone considers encapsulating cells into the selected biomaterial to prepare the bioink [30]. Our earlier published work explored the impact of applied printing pressures dispensing through nozzles having two different diameters [22]. We demonstrated that extruding with the same printing pressure through a smaller nozzle diameter resulted in a higher dead cell count close to the nozzle wall compared to a larger nozzle. One of the required rheological properties of hydrogel being used in extrusion-based bioprinting is shear thinning behavior [31]. Reduction of viscosity or increment of shear stress with increasing shear rate is termed as shear thinning behavior [3]. High viscous biomaterials encapsulating living cells experiences higher shear stress deposition through a nozzle, which negatively affects the encapsulated cells. Therefore, hydrogel having shear thinning behavior with high recoverability is essential in the extrusion-based 3D bioprinting technique to provide comfort to encapsulated cells and maintain geometric fidelity. To analyze the shear thinning behavior of hydrogels, the Power-Law Equation (Equation (1)), specifically shear thinning co-efficient of *n* and *K*, plays an import role [32]. The value of *n* equal to 1 indicates Newtonian fluid where *n* < 1 and *n* > 1 express pseudoplastic and dilatant fluids, respectively [33,34].

Lower lateral dimensions of nanofibrils and high carboxylate content make TO-NFC less light-scattering and introduce better electrostatic stabilization by obstructing aggregation [35]. Many studies have been conducted to identify the impact of TO-NFC and cellulose nanocrystals (CNCs) on the rheological properties of the suspensions [36,37]. Various inorganic acids such as sulfuric and hydrochloric acids were utilized to improve the aspect ratio and dispersibility of NFC and CNC. However, sulfuric acid can lead to cellulose esterification, resulting in lower thermal stability. On the other hand, functionalization may not occur on the nanocellulose surface, which can lead to control of the colloidal stability of NFC or CNC in aqueous solution [38].

In our earlier research, we developed a novel bioink that comprised alginate, carboxymethyl cellulose (CMC), and TEMPO-mediated nano-fibrillated cellulose (TO-NFC) limiting the solid content to 5% (2% alginate, 2% CMC, and 1% TO-NFC) that ensured fabrication of a scaffold with a build height of 9.6 mm and 93% cell viability [22]. As an extension of our earlier work, in this paper we explored the effect of a very low and high percentage of TO-NFC on varying ratios of alginate and CMC in terms of flow behavior. An appropriate selection of TO-NFC percentage to control the flow behavior (i.e., co-efficients of *n* and *K*) can help select composition for an extrusion-based 3D bioprinting process to fulfill any specific application. Since this research deeply explores the values of *n* and *K* for compositions having various percentages of TO-NFC and investigates the relations between them, it can help select material compositions that require lower applied pressure to extrude and maintain a good shape fidelity of the fabricated scaffolds using the resulted values of *n* and *K*. We will demonstrate here how the *n* and *K* values can be controlled with various bio-material compositions and their consequent effects on 3D printability and shape fidelity of fabricated filaments. Extensive rheological experiments were conducted on a big sample size such as 46 compositions prepared with varying alginate, CMC, and TO-NFC percentages. The changes of rheological behavior, specifically the flow behavior of those compositions, were co-related with various percentages of TO-NFC. Finally, we chose some compositions having very low and high percentages of TO-NFC to a 3D print filament, analyzed the shape fidelity, and co-related the result.

## 2. Materials and Methods

### 2.1. Processing of TO-NFC and Hydrogel Preparation

**Figure 1:**
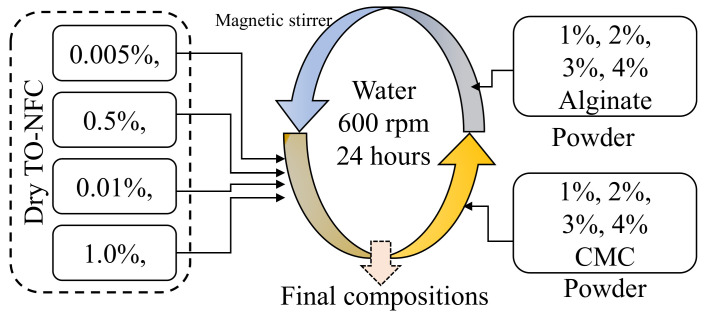
Schematic representation of the preparation of compositions.

Dry TEMPO nano-fibrillated cellulose (TO-NFC) [(C_6_H_10_O_5_)_x_(C_6_H_9_O_4_CO_2_Na)_y_] with a carboxylate level from 0.2 to 2 mmol/g solids was acquired from the Process Development Center (PDC) at the University of Maine. Four different percentages of dry TO-NFC such as 0.005%, 0.01%, 0.5%, and 1.0% (*w*/*v*) were prepared using a magnetic stand-up stirrer with 600 rpm for 24 h at room temperature. Various percentages of medium (viscosity ≥2000 cps of 2% in water) viscous alginate (1, 2, 3, and 4%, *w*/*v*) and CMC (1, 2, 3, and 4%, *w*/*v*) (pH: 6.80) (Sigma-Aldrich, St. Louis, MO, USA) were mixed with prepared TO-NFC with a magnetic stand-up stirrer to make a uniform composition. An overview of preparing the hybrid hydrogel is shown in Figure 1. Compositions used in this paper to prepare the bioink are shown in Table 1 and Table 2 where letters ‘A’, ‘C’, and ‘T’ represent Sodium alginate, Carboxymethyl cellulose, and tempo-mediated Nano fibrillated cellulose, respectively. The numerical subscripts represent the weight percentage of the components mixed into the water to prepare the material compositions. The addition of CMC and TO-NFC will increase the overall viscosity of the material, which will assist in achieving better printability and shape fidelity.

### 2.2. Rheological Analysis 

We conducted the rheological tests with a rotational rheometer (MCR 102, Anton Paar, Graz, Austria) having a parallel plate geometry (25.0 mm flat plate). The plate-to-plate gap was maintained at 1.0 mm, and all data were recorded at room temperature (25 °C) having the intention that the extrusion process will be performed at room temperature to have a quick gelation of the deposited filament [39]. We mainly focused on the flow behavior of the considered compositions. For flow curve analysis, a steady-rate sweep test was conducted having a variable shear strain from 0.1 to 100 s^−1^. To analyze the shear thinning behavior of considered compositions, the Power-Law Equation (Equation (1)) was fitted to the linear region of the shear strain rate vs. viscosity curve [32]. Then the shear thinning co-efficients of *n* and *K* were determined by fitting a curve to the following equation:

(1) $$\eta =K{\dot{\gamma }}^{n-1}$$

where η is the viscosity and $\dot{\gamma }$ is the shear rate (SR). While the material is extruded through the nozzle, shear stress occurs throughout the material and is larger along the nozzle wall. A nonlinear curve-fitting module (Allometric) of OriginPro 2022b (Originlab, Northampton, MA, USA) was used to fit the viscosity vs. shear rate data for each composition to determine the values of *n* and *K*.

The specific shear stress driving the solid-like state to a liquid-like state to initiate the flow is termed as yield stress. This behavior can be described with the following Herschel–Bulkley equation [40]:

(2) $$\tau =\tau_{0}+K{\dot{\gamma }}^{n}$$

where $\tau_{0}$ is yield stress [41] and τ is dynamic/apparent shear stress with respect to the shear rate.

### 2.3. 3D Printing and Shape Fidelity Analysis

We used an extrusion-based 3D bio-printer [BioX (CELLINK, Boston, MA, USA)] to fabricate the filaments and scaffolds. We prepared hydrogels, accordingly, loaded them into a 3.0 mL disposable nozzle, and extruded them pneumatically on a stationary build plane. The printing parameters we used in fabricating the scaffolds were a nozzle diameter of 700 µm; print speed of 5, 10, and 15 mm/s; and air pressure varying from 27–110 kPa as shown in Table 1 and Table 2. To fabricate multilayered scaffolds (20 mm × 20 mm), we used a nozzle with 410 µm diameter, 8 mm/s print speed, 110 kPa air pressure, and two different porosities (5% and 15%). A visual basic-based Computer-Aided Design (CAD) software, Rhino 6.0 (https://www.rhino3d.com, accessed on 10 November 2022), was used to design and define the vectorized toolpath of a scaffold. Slicer (https://www.slicer.org, accessed on 10 November 2022), a G-code generator V1.50 software, was used to generate a Bio-X-compatible file including the toolpath coordinates and all process parameters to build the scaffold. We followed a layer-upon-layer fashion to release the materials. The images of fabricated filaments were captured using the CK Olympus bright field microscope [42]. The width of the filament is determined using ImageJ software.

### 2.4. Investigation on Microstructure and Biocompatibity

The prepared TO-NFC slurry was stored at 4 °C before further analysis. This slurry was air dried to prepare a thin film with a thickness of 100 μm. A JEOL JSM-7600F scanning electron microscope (JEOL USA, Peabody, MA, USA) operating at 2 kV was used to capture a set of images of this thin film. The SEM images of the TO-NFC film demonstrate the fiber distribution, whereas the SEM image of the filament composition of alginate, CMC, and TO-NFC reveal the microstructure.

To investigate the biocompatibility of fiber-filled biomaterials, we encapsulated Porc 1 airways smooth muscle cell (passage four) with A_2_C_2_T_1_ to prepare the bioink following our protocol published earlier [22]. In short, Porc1 cells were cultured and maintained in high glucose DMEM/F12, 10% fetal bovine serum (FBS), with 100 μg/mL penicillin, and 100 μg/mL streptomycin (Sigma-Aldrich) in a 5% CO_2_ and 37 °C incubator. Around 12 × 10^6^ cells were re-suspended in 200 μL of culture medium before being mixed with 1.0 mL sterile hydrogel solution. The cells were mixed uniformly with a magnetic stirrer at a very low speed to obtain the final cell concentration of 10 × 10^6^ cell/mL in our cell-laden bioink. Scaffolds were printed with the bioink and cross-linked with CaCl_2_, washed three times with Hanks’ balanced salt solution (HBSS), and finally incubated in fresh media at 37 °C, 5% CO_2_, and more than 90% humidity. The cell viability was determined by live/dead assay using calcein green AM and propidium iodide (Thermofisher, Waltham, MA, USA)

### 2.5. Statistical Analysis

We collected data following a format of “mean ± standard deviation” and analyzed them using a significance level of *p* = 0.05 with a two-way ANOVA with 95% confidence interval. Calculations were performed with *n* = 3 unless otherwise stated. We used a statistical software, Origin Pro 2021b, to analyze quantitatively and graphically.

## 3. Results

Rheological properties of the compositions were determined especially in terms of their viscosities. The shear thinning behavior was accessed through the Flow Curve test on all the compositions. This revealed the impact of various concentrations of the nanofibers in the viscosities of different compositions portrayed via graphs given below:

### 3.1. Flow Behavior of Hydrogels Prepared by 0.005% and 0.5% TO-NFC

**Figure 2:**
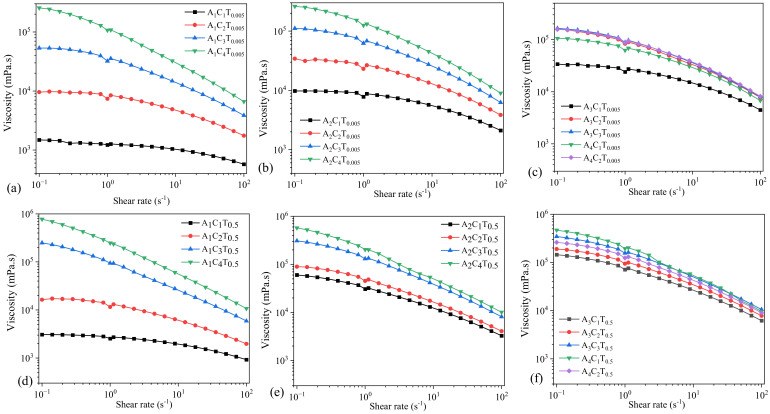
Viscosity of compositions composed with various percentages of alginate (1–4%) and CMC (1–4%) with (**a**–**c**) 0.005% of TO-NFC and (**d**–**f**) 0.5% of TO-NFC.

With the use of 0.005% of TO-NFC, all the compositions with varying concentrations of alginate and CMC showed a shear thinning behavior. A_1_C_1_T_0.005_ showed the least viscosity while A_1_C_4_T_0.005_ and A_2_C_4_T_0.5_ showed the highest and similar viscosities. Results are shown in Figure 2. Similarly, A_1_C_1_T_0.5_ lay at the lowest spectrum of the viscosity while A_1_C_4_T_0.5_ showed the highest viscosity. Also, when T_0.005_ and T_0.5_ are compared, compositions with T_0.5_ have higher viscosities than the compositions mixed with T_0.005_. This concludes that the higher the concentration of the TO-NFC, the higher the viscosity of the composition will be when the composition of alginate and CMC are kept constant.

**Figure 3:**
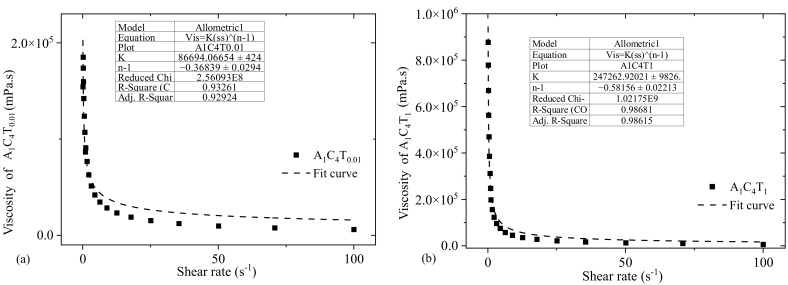
Estimating the values of shear thinning factors (*n* and *K*) of compositions (**a**) A_1_C_4_T_0.01_ and (**b**) A_1_C_4_T_1_. For A_1_C_4_T_0.01_, *n* and *K* values were 0.64 and 86694 ± 424 (mPaS) with an R-square value of 0.929, whereas, for A_1_C_4_T_1_, *n* and *K* values were 0.42 and 247262 ± 9826 (mPaS) with an R-square value of 0.9868.

**Figure 4:**
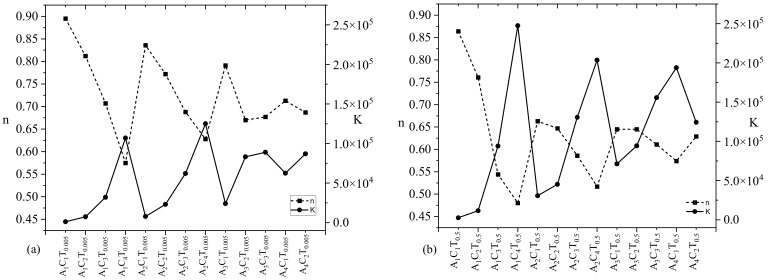
Shear thinning factors (*n* and *K*) of compositions composed with various percentages of alginate (1–4%) and CMC (1–4%) with a constant percentage (**a**) 0.005% and (**b**) 0.5% of TO-NFC. All *n* values less than 1 confirm a shear thinning behavior of all compositions.

As mentioned in Section 2.2, a nonlinear curve fitting module (Allometric) was used to fit the viscosity vs. shear rate data for each composition to determine the values of *n* and *K*. For each composition, adjusted R-square value of more than 90% implies a good fit. For an example, to determine the *n* and *K* values of A_1_C_4_T_0.01_ and A_1_C_4_T_1_, the fit curves are shown in Figure 3. Adjusted R-square values for the curves fitted for A_1_C_4_T_0.01_ and A_1_C_4_T_1_ compositions were 92.9% and 98.6%, respectively, as shown in Figure 4. Similarly, the *n* and *K* values for all other compositions were determined in this paper. Upon the calculation of the shear thinning factors, for all n and k of the compositions shown in Figure 3, we found *n* < 1, which implies all have shear thinning behavior. The value of *K* increases with the increase in the concentration of TO-NFC.

Figure 4 also depicts an interesting phenomenon: irrespective of percentage of TO-NFC (either 0.005% or 0.5%), the *n* and *K* values showed an inverse relation. The value of *n* close to 1.0 means the hydrogel has viscosity similar to water. Lower *n* values represent higher shear thinning capacity (i.e., tendency of higher viscosity reduction with a small increment of the shear rate on the hydrogel). For an example, the *n* values for A_1_C_1_T_0.005_ and A_1_C_1_T_0.5_ are 0.90 and 0.86 where the *K* values are 1222 and 2535 mPa.s, respectively, as shown in Figure 4. For both compositions, the *n* and *K* values are inversely related. Either *n* or *K* values confirm that A_1_C_1_T_0.5_ is more viscous than A_1_C_1_T_0.005_.

The shear thinning factor *K* was determined at the shear rate 1.0 s^−1^. For comparison purposes, we will mostly use the *n* and *K* values throughout the paper. With a constant percentage of TO-NFC (either 0.005% or 0.5%) and alginate (either 1%, or 2%, or 3%, or 4%), the viscosity was dictated by the percentage of CMC. As an example: A_1_C_4_T_0.005_ showed highest amount of *K* value among A_1_C_1_T_0.005_, A_1_C_2_T_0.005_, A_1_C_3_T_0.005_, and A_1_C_4_T_0.005_. A similar phenomenon was observed for A_2_C_4_T_0.005_, A_3_C_3_T_0.005_, and A_4_C_2_T_0.005_ as shown in Figure 2. However, for a similar percentage of solid content (i.e., summation of percentage of all components into a specific composition), CMC may not control the *K* value all the time. As an example, the *K* values for A_1_C_4_T_0.005_, A_2_C_3_T_0.005_, A_3_C_2_T_0.005_, and A_4_C_1_T_0.005_ were 106999, 62205, 83266, and 62491 mPa.s, respectively. Here, the summation of solid content for each composition is 5.005%. Composition (A_1_C_4_T_0.005_) having a 4% CMC showed highest *K* value. The data indicates that the *K* value did not follow a trend with the percentage of CMC.

Even with increasing the percentage of TO-NFC from 0.005% to 0.5%, a comparable scenario was also observed for the *K* values of A_1_C_4_T_0.5_, A_2_C_3_T_0.5_, A_3_C_2_T_0.5_, and A_4_C_1_T_0.5_ (247426, 130574, 94258, and 193857 mPa.s, respectively) as shown in Figure 2. Here, the summation of solid content for each composition is 5.5%, and the percentage of CMC did not fully govern the *K* value.

### 3.2. Flow Behavior of Hydrogels Prepared by 0.01% and 1.0% TO-NFC

**Figure 5:**
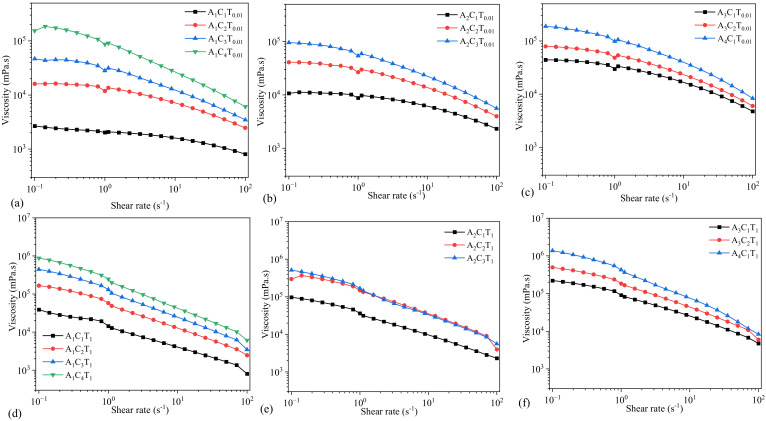
Viscosity of compositions composed with various percentages of alginate (1–4%) and CMC (1–4%) with (**a**–**c**) 0.01% of TO-NFC and (**d**–**f**) 1.0% of TO-NFC.

We increased the percentage of TO-NFC from 0.005% to 0.01% and 0.5% to 1.0% to analyze the changes in viscosity and related shear thinning factors. From Figure 4, A_1_C_1_T_0.01_ showed significantly less viscosity than any of the compositions while A_4_C_1_T_0.01_ had the highest viscosity of all. Similarly, A_1_C_1_T_1_ projected the smallest viscosity while A_1_C_4_T_1_ showed the highest (Figure 5). The changes in the concentration of TO-NFC had a slight impact on the viscosity of compositions as compared to when the concentrations were changed from 0.005% to 0.5%. The value of *n* < 1 demonstrates that they all show shear thinning properties. The value of *K* within constant concentration of TO-NFC and alginate depicts that the viscosity also heavily depends upon the concentration of CMC, which is used as a viscosity thickener.

However, as discussed earlier, for a similar percentage of solid content, CMC may not control the *K* value all the time. For an example, the *K* values for A_1_C_4_T_0.01_, A_2_C_3_T_0.01_, A_3_C_2_T_0.01_, and A_4_C_1_T_0.01_ were 86694, 53915, 48302, and 98458 mPa., respectively. Here, the summation of solid content for each composition is 5.01%. A composition (A_1_C_4_T_0.01_) having a 4% CMC showed the highest *K* value. Outlined data clarifies that *K* value did not follow a trend with the percentage of CMC. Even a similar phenomenon was observed with 1% TO-NFC leaving the percentages of alginate and CMC unchanged. The *K* values for A_1_C_4_T_1_, A_2_C_3_T_1_, A_3_C_2_T_1_, and A_4_C_1_T_1_ were 247785, 166808, 184119, and 424153 mPa.s, which did not follow any trend with the percentage of CMC.

**Figure 6:**
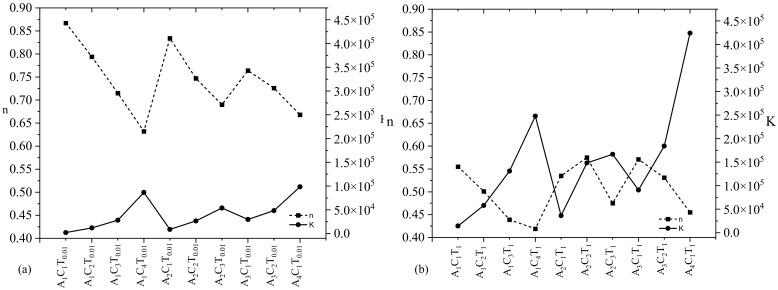
Shear thinning factors (*n* and *K*) of compositions composed with various percentages of alginate (1–4%) and CMC (1–4%) with a constant percentage (**a**) 0.01% and (**b**) 1.0% of TO-NFC. All *n* values less than 1 confirm a shear thinning behavior of all compositions.

Figure 6 follows a similar trend to Figure 4 where the *n* and *K* values showed an inverse relation irrespective of percentage of TO-NFC (either 0.01% or 1.0%). Lower *n* value represents the tendency of higher viscosity reduction with a small increment of the shear rate on the hydrogel. As a proof, the *n* values for A_1_C_1_T_0.01_ and A_1_C_1_T_1_ are 0.87 and 0.55 where the *K* values are 2034 and 14422 mPa.s, respectively, as shown in Figure 6. Therefore, the *n* and *K* values are inversely related for both compositions and confirm A_1_C_1_T_1_ is more viscous than A_1_C_1_T_0.01_.

### 3.3. Impact of Higher Percentages of TO-NFC over Lower Percentages of TO-NFC

**Figure 7:**
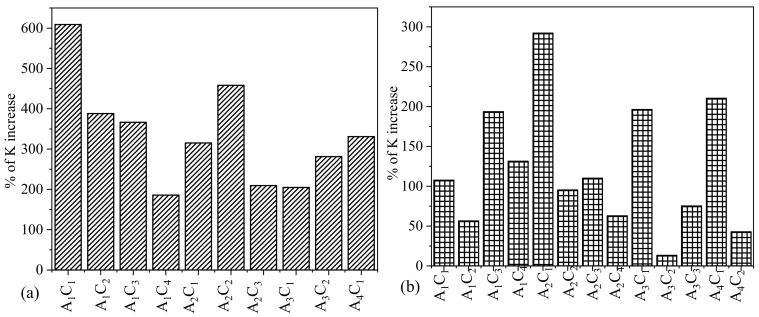
Percentage of *K* (viscosity at 1.0 s^−1^ shear rate) increase of compositions having (**a**) 1% TO-NFC compared to 0.01% TO-NFC and (**b**) 0.5% TO-NFC compared to 0.005% TO-NFC.

In our earlier work, we demonstrated that the presence of TO-NFC can significantly improve the overall viscosity of the composition having less amount of solid content [22]. A_2_C_2_T_1_, which has 5% solid content, turned out to be able to fabricate large-scale scaffolds having 42 layers using relatively lower applier pressure (10 psi). This might happen because of increasing the rate of cross-link due to the presence of more polar carbonyl group (C^δ+^=O^δ−^), which drives toward a high rate of cross-linking. In this paper, we explored the impact of various percentages of TO-NFC on the flow behavior of the compositions. We used a total of four different weight percents of TO-NFC such as 0.005%, 0.01%, 0.5%, and 1.0% (*w*/*v*) with various percentages of alginate (1–4%) and CMC (1–4%). Two of them are at the lower end such as 0.005% and 0.01%, and the rest of them are at the higher end such as 0.5% and 1.0%. In both cases, we increased the TO-NFC amount to 100% such as 0.005% to 0.5% and 0.01% to 1.0% to realize its impact. We demonstrated the % of K increase of A_1_C_1_T_1_, A_1_C_2_T_1_, A_1_C_3_T_1_, A_1_C_4_T_1_, A_2_C_1_T_1_, A_2_C_2_T_1_, A_2_C_3_T_1_, A_3_C_1_T_1_, A_3_C_2_T_1_, and A_4_C_1_T_1_ with respect to the same composition of alginate and CMC prepared with 0.01% of TO-NFC as shown in Figure 7a. We observed a range of the percentage of K increase from 205% (A_1_C_4_T_1_) to 609% (A_1_C_1_T_1_) compared to the same composition of alginate and CMC prepared with 0.01% of TO-NFC (such as A_1_C_4_T_0.01_ and A_1_C_1_T_0.01_). Increasing TO-NFC 100% from 0.01% to 1.0% increases the cross-linking density into each composition due to more availability of the carbonyl ion (–COO–). Moreover, the hydrogen bonds between the same carbonyl ion make the composition stronger prepared with 1.0% of TO-NFC.

We also demonstrated the similar behavior of % of *K* increase of A_1_C_1_T_0.5_, A_1_C_2_T_0.5_, A_1_C_3_T_0.5_, A_1_C_4_T_0.5_, A_2_C_1_T_0.5_, A_2_C_2_T_0.5_, A_2_C_3_T_0.5_, A_2_C_4_T_0.5_, A_3_C_1_T_0.5_, A_3_C_2_T_0.5_, A_3_C_3_T_0.5_, A_4_C_1_T_1_, and A_4_C_2_T_1_ with respect to the same composition of alginate and CMC prepared with 0.005% of TO-NFC as shown in Figure 7b. We observed a range of the percentage of *K* increase from 13.2% (A_3_C_2_T_0.5_) to 292% (A_2_C_1_T_0.5_) compared to the same composition of alginate and CMC prepared with 0.01% of TO-NFC (such as A_3_C_2_T_0.005_ and A_2_C_1_T_0.005_). Since, 1% TO-NFC provides more sites to form internal cross-linking compared to 0.5% TO-NFC, we observed a higher change of percentage of *K* increase for the compositions having 1% TO-NFC. Increasing TO-NFC 100% from 0.005% to 0.5% increases the cross-linking density into each composition due to the higher availability of carbonyl ion (–COO–). The hydrogen bonds between the same carbonyl ion make the composition stronger prepared with 0.5% of TO-NFC.

### 3.4. Dynamic Shear Stress and Yield Stress of Compositions Prepared with 0.005%, 0.01%, 0.5%, and 1.0% TO-NFC

**Figure 8:**
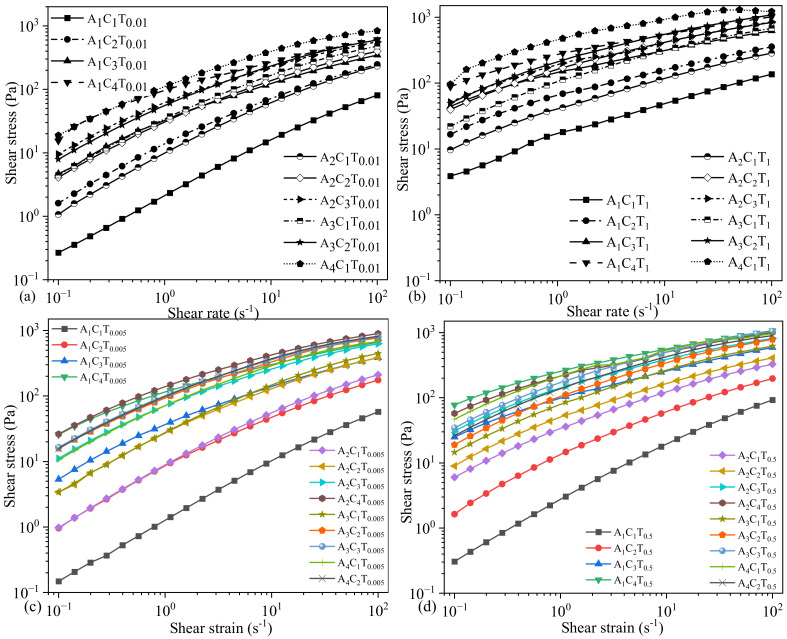
Shear stress of compositions prepared by (**a**) 0.01%, (**b**) 1.0%, (**c**) 0.005%, and (**d**) 0.5% TO-NFC. These results also showed a shear thinning behavior where the shear stress was increased with increasing the shear rate. It basically followed the Herschel–Bulkley equation [40], $\tau =\tau_{0}+K{\dot{\gamma }}^{n}$ where $\tau_{0}$ is yield stress [41] and τ is dynamic shear stress with respect to the shear rate.

Shear stresses were recorded at various shear rates for all the compositions mentioned in Table 1 and Table 2 where they showed an increasing trend of shear stress with increasing the shear rate meaning the transition from a solid-like state to liquid-like state. The specific shear stress driving the solid-like state to liquid-like state to initiate the flow is termed as yield stress ($\tau_{0}$). This behavior was described with the Herschel–Bulkley equation (Equation (2)). As we proved in Section 3.1 and Section 3.2 with the shear thinning factors *n* and *K*, all the compositions we considered in this paper showed shear thinning behavior; Figure 8 echoed the same phenomenon with shear stress vs. shear rate plots. However, shear stresses were significantly controlled by the amount of TO-NFC. As an example, the shear stresses for a composition A_4_C_1_ mixed with various percentages of TO-NFC such as 0.01% (A_4_C_1_T_0.01_), 0.5% (A_4_C_1_T_0.5_), and 1.0% (A_4_C_1_T_1_) showed 15.7% (118 Pa), 121% (226 Pa), and 366% (475.56 Pa) higher shear stress at 1.12 s^−1^ shear rate compared to the composition mixed with 0.005% (102 Pa) TO-NFC (A_4_C_1_T_0.005_). A similar phenomenon was observed for other compositions as well, as shown in Figure 8.

After fitting the shear stress plot with Equation (2), considering the *K* and *n* values equal resulted from power law (Equation (1)), the yield stresses (that converted the solid-like state to liquid-like state to initiate the flow), and corresponding shear rates were determined as shown in Table 3. Data indicates that the amount of CMC controlled YS for a constant amount of TO-NFC and alginate. Likewise, for a constant amount of alginate and CMC, the YS was changed depending on the amount of TO-NFC. Moreover, the required amount of stress created the smooth fluid flow, i.e., YS was dependent on solid content percentage . To achieve that required amount of YS, different amounts of the applied shear rate were needed as shown in Table 3. As an example, we determined the YSs 100.42 Pa, 111.49 Pa, 46.80 Pa, and 302.48 Pa for the compositions of A_2_C_3_T_0.005_, A_2_C_3_T_0.5_, A_2_C_3_T_0.01_, and A_2_C_3_T_1_, respectively, where each of the compositions has the solid content as 5.005%, 5.5%, 5.01%, and 5.1%, respectively. However, to achieve these amounts of YSs, A_2_C_3_T_0.005_, A_2_C_3_T_0.5_, A_2_C_3_T_0.01_, and A_2_C_3_T_1_ needed 1.12 s^−1^, 1.12 s^−1^, 0.794 s^−1^, and 2.24 s^−1^ amount of shear rates, respectively.

### 3.5. Amplitude Test: Storage and Loss Modulus

**Figure 9:**
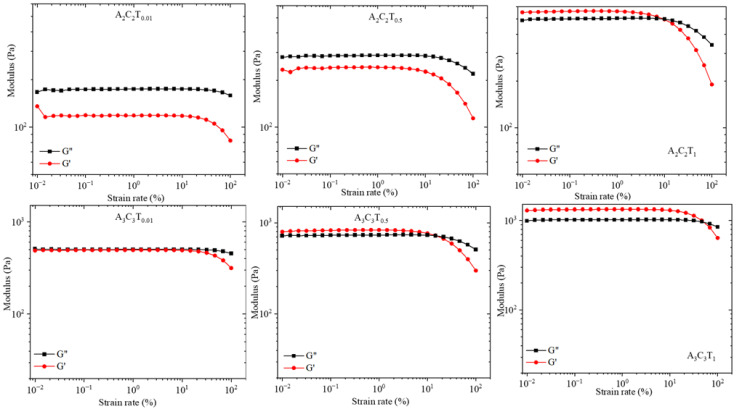
Loss modulus (G″) and storage modulus (G′) of A_2_C_2_T_0.01_, A_2_C_2_T_0.5_, A_2_C_2_T_1_, A_3_C_3_T_0.01_, A_3_C_3_T_0.5_, and A_3_C_3_T_1_ resulting from amplitude sweep test. Any composition shows G″ > G′ (such as A_2_C_2_T_0.01_, A_2_C_2_T_0.5_, and A_3_C_3_T_0.01_) represents liquid-like character where G′ > G″ (such as A_2_C_2_T_1_, A_3_C_3_T_0.5_, and A_3_C_3_T_1_) indicates solid-like character.

As a representative, we considered a subset of compositions such as A_2_C_2_T_0.01_, A_2_C_2_T_0.5_, A_2_C_2_T_1_, A_3_C_3_T_0.01_, A_3_C_3_T_0.5_, and A_3_C_3_T_1_ to conduct the amplitude sweep test. This test was conducted at 1 Hz, and the outcome of loss modulus (G″) and storage modulus (G′) versus shear strain rate (%) were plotted as shown in Figure 9. Figures depict that higher percentages of TO-NFC and overall solid content drive the physical state of composition from a liquid-like to a solid-like state. Any composition having higher loss modulus (G″) dictates the liquid-like state within the strain rate used. In Figure 9, we observe A_2_C_2_T_0.01_, A_2_C_2_T_0.5_, and A_3_C_3_T_0.01_ compositions showed higher G″ compared to G′. The term dynamic mechanical loss tangent (tan δ = G″/G′, δ is the phase the angle) is also used where tan δ > 1 indicates the liquid-like state. With increasing the percentage of TO-NFC from 0.01% to 1.0%, we observed the shifting of the physical state from liquid-like to solid-like where G′ started dominating G″ and resulted in tan δ < 1. Compositions A_2_C_2_T_1_, A_3_C_3_T_0.5_, and A_3_C_3_T_1_ dictated a solid-like character with higher G′ value. For these three compositions, G’ dominated G″ up to a particular strain-rate level and then intersected (G′ = G″). That limit is called the Linear Viscosity Range (LVR), and it indicates the limit at which suspension preserves the sedimentation within the sample without permanent deformation [43]. We observed LVR at 10%, 14.7%, and 55% of strain rate for A_2_C_2_T_1_, A_3_C_3_T_0.5_, and A_3_C_3_T_1_, respectively, indicating that higher solid content increases the dominancy of G′. Compositions with a higher value of G′ before reaching the intersecting point with G″ pose good mechanical strength with highly structured solid-like behavior of the suspension. This character can be used in 3D bioprinting process to fabricate scaffolds with defined geometry [44,45].

### 3.6. Three Point Thixotropic Test (3iTT)

**Figure 10:**
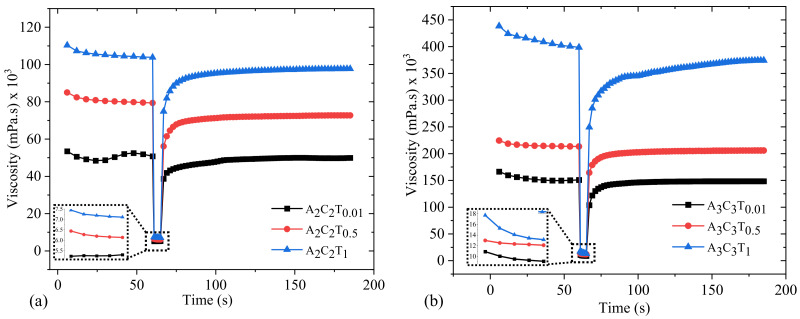
Three-point thixotropic test for the compositions of (**a**) A_2_C_2_T_0.01_, A_2_C_2_T_0.5_, and A_2_C_2_T_1_ and (**b**) A_3_C_3_T_0.01_, A_3_C_3_T_0.5_, and A_3_C_3_T_1_. It clearly shows that higher shear rate breaks the internal bonds of the composition, makes it easier to flow through the nozzle during extrusion, and recovers its internal bonds after releasing from the nozzle.

Typically, in extrusion-based 3D bioprinting, stress is applied to the at-rest hydrogel, which breaks down the initial network structures of the hydrogel. After the hydrogel is extruded through the nozzle at a specific shear rate, the initial internal network of the hydrogel is reformed. To mimic the extrusion-based 3D bioprinting scenario, we conducted a three-point thixotropic test on a subset of compositions such as A_2_C_2_T_0.01_, A_2_C_2_T_0.5_, A_2_C_2_T_1_, A_3_C_3_T_0.01_, A_3_C_3_T_0.5_, and A_3_C_3_T_1_. Figure 10 shows that shear rate was used 1.0 s^−1^ for 60 s, was abruptly increased to 100s^−1^ for 5 s, and finally, was reduced to 1.0 s^−1^ for rest of 120 s. Right after releasing the higher shear rate, A_2_C_2_T_0.01_, A_2_C_2_T_0.5_, and A_2_C_2_T_1_ showed recovery rates of 76%, 71%, and 72%, respectively. The recovery rates increased with time. For example, the recovery rates for A_2_C_2_T_0.01_, A_2_C_2_T_0.5_, and A_2_C_2_T_1_ increased up to 91%, 88%, and 90%, respectively, after 30 s of releasing the shear rate. We observed similar behavior for the compositions of A_3_C_3_T_0.01_, A_3_C_3_T_0.5_, and A_3_C_3_T_1_. They showed 68%, 77%, and 62% recovery rates right after releasing the shear rate. The recovery rate increased with time. The scenario applying a higher shear rate is shown in insets of Figure 10.

### 3.7. Analysis of Filament Width after 3D Printing

**Figure 11:**
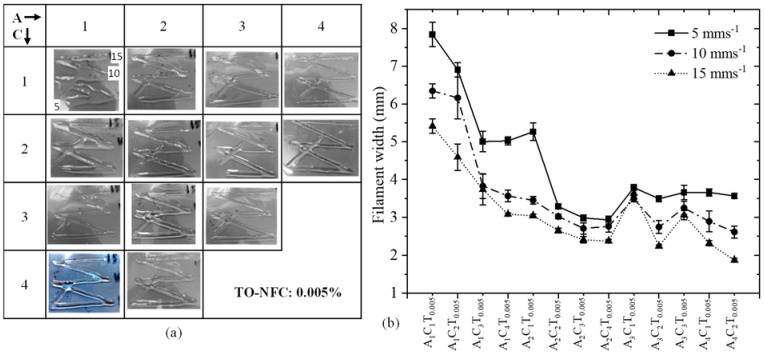
(**a**) Fabricated filaments with 1%, 2%, 3%, and 4% alginate; 1%, 2%, 3%, and 4% CMC; and 0.005% TO-NFC. (**b**) Filament widths of all those compositions.

**Figure 12:**
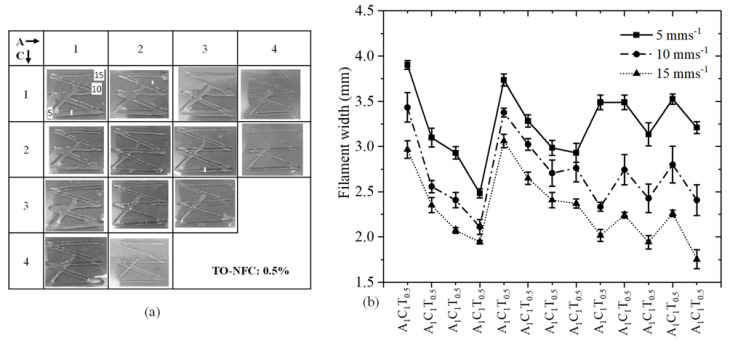
(**a**) Fabricated filaments with 1%, 2%, 3%, and 4% alginate; 1%, 2%, 3%, and 4% CMC; and 0.5% TO-NFC. (**b**) Filament widths of all those compositions.

**Figure 13:**
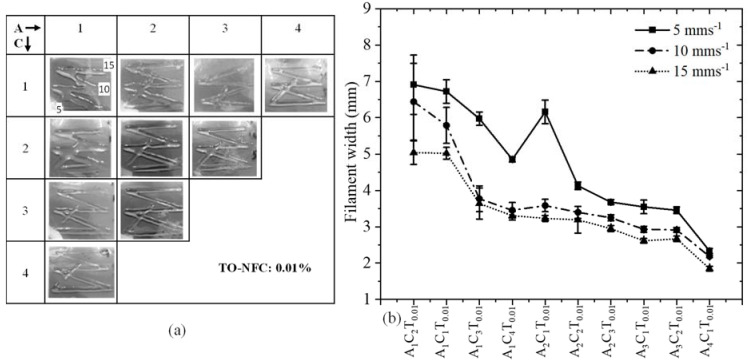
(**a**) Fabricated filaments with 1%, 2%, 3%, and 4% alginate; 1%, 2%, 3%, and 4% CMC; and 0.01% TO-NFC. (**b**) Filament widths of all those compositions.

**Figure 14:**
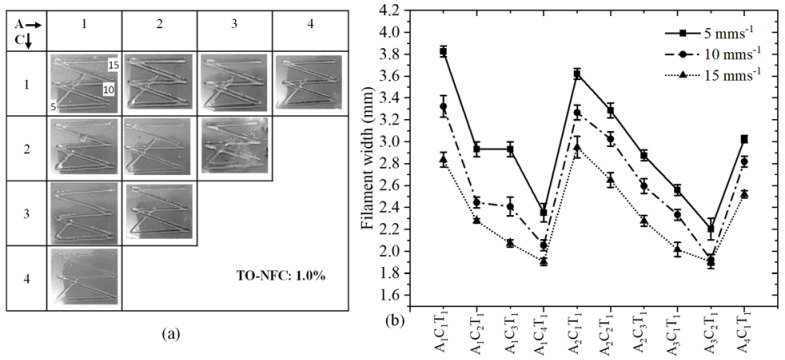
(**a**) Fabricated filaments with 1%, 2%, 3%, and 4% alginate; 1%, 2%, 3%, and 4% CMC; and 1.0% TO-NFC. (**b**) Filament widths of all those compositions.

To analyze the effect of TO-NFC on the printability of various compositions having 0.005%, 0.5%, 0.01%, and 1.0% TO-NFC, 3D prints were considered as shown in Figure 11a, Figure 12a, Figure 13a and Figure 14a. With three different print speeds such as 5, 10, and 15 mm/s, we printed all compositions. Our first observation was all compositions were 3D-printable and most of the filaments preserved the shape. Filament diameters for each composition were determined as shown in Figure 11b, Figure 12b, Figure 13b and Figure 14b. All figures depict that with increasing the percentage of TO-NFC, the filament geometry was better defined with smaller deviation from the nozzle diameter, i.e., less spread of hydrogels. For any composition, the filament width was reduced with increasing print speed. As an example, filament width for composition A_1_C_4_T_0.005_ was reduced up to 21.2% for print speed of 10 mms^−1^ and 30.5% for print speed of 15 mms^−1^ compared to the filament width printed with 5 mms^−1^. A similar scenario was observed for other compositions as shown in Figure 11b, Figure 12b, Figure 13b and Figure 14b. It also indicates that filaments fabricated with 1% TO-NFC showed lower randomness or a better trend compared to the filaments fabricated with 0.005%, 0.01%, and 0.5% TO-NFC.

Filaments fabricated with lower concentrations of TO-NFC showed larger width. As shown in Table 3, the yield stresses (0.28 Pa and 0.35 Pa) and corresponding shear rates (0.199 s^−1^ and 0.141 s^−1^) for A_1_C_1_T_0.005_ and A_1_C_1_T_0.01_ were significantly smaller than other compositions. The shear rate produced with the applied pressure was substantially higher, and it resulted in higher shear stress compared to yield stress, which finally drove releasing additional materials and ending up with a larger filament width. However, with increasing the percentage of TO-NFC, yield stresses and corresponding shear rates increased, meaning compositions needed higher applied pressure to release material through the nozzle. This additional shear stress can negatively affect the encapsulated cells during extrusion.

**Figure 15:**
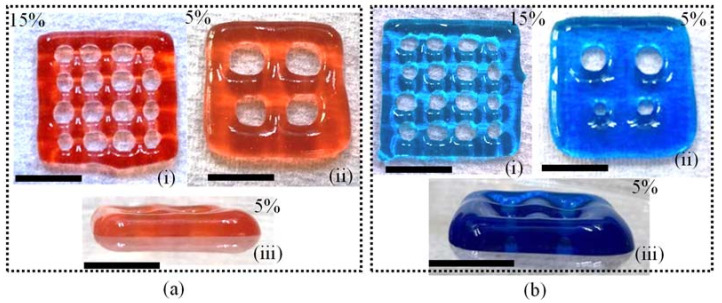
Scaffolds fabricated with (**a**) A_3_C_3_T_0.01_ and (i) 15% porosity, (ii) 5% porosity, (iii) lateral view of the scaffold having 5% porosity; (**b**) A_3_C_3_T_1_ and (i) 15% porosity, (ii) 5% porosity, (iii) lateral view of the scaffold having 5% porosity. Scale bar = 10 mm.

Finally, we used two compositions such as A_3_C_3_T_0.01_ and A_3_C_3_T_1_ to fabricate a set of scaffolds having three and eight layers as shown in Figure 15a,b, respectively. We used a porosity of 15% to print three layers and a porosity of 5% to print eight layers. Figures indicate that A_3_C_3_T_1_ has a lower tendency to spread than A_3_C_3_T_0.01_ resulting in better_-_defined scaffold structures. However, in the future, we will explore the 3D printability of other compositions mentioned in this paper with various nozzle diameters, applied pressures, and print speeds.

## 4. Discussion of Microstructure and Biocompatibility

**Figure 16:**
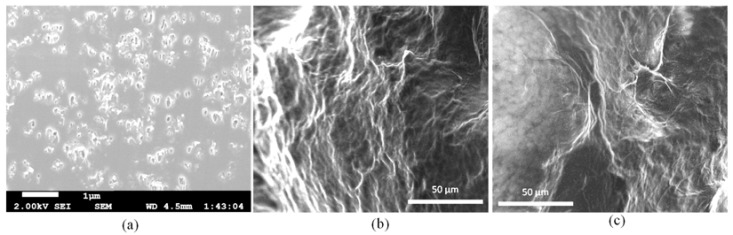
(**a**) SEM image of the pure TO-NFC film of 100 μm. It represents the uniform distribution of TO-NFC fiber. SEM imaging performed on the cross-section of filaments fabricated with (**b**) A_2_C_2_T_1_ and (**c**) A_2_C_2_T_0.5_.

The cross-sectional view of the thin dried film shown in Figure 13a demonstrates the fiber distribution. Fibers were distributed randomly and uniformly into the film and did not coagulate. To analyze the microstructure morphologically, SEM imaging was also conducted on the compositions of A_2_C_2_T_1_ and A_2_C_2_T_0.5_, as shown in Figure 16b,c, respectively. A homogeneous distribution of alginate, CMC, and TO-NFC along with smooth cell structure was observed. This homogeneous distribution may result from the strong physical interaction between alginate, CMC, and TO-NFC. We also investigated the swelling rate of filaments fabricated with A_2_C_2_T_1_ and A_2_C_2_T_0.5_ in our previous article [22] where we reported 360% and 277% swelling rates, respectively, after 10 incubation days. Pore closer rate for scaffolds fabricated with A_2_C_2_T_1_ and A_2_C_2_T_0.5_ showed 55% and 51%, respectively, in that paper. In the future, we plan to determine the swelling rate for other compositions that are mentioned in this paper.

**Figure 17:**
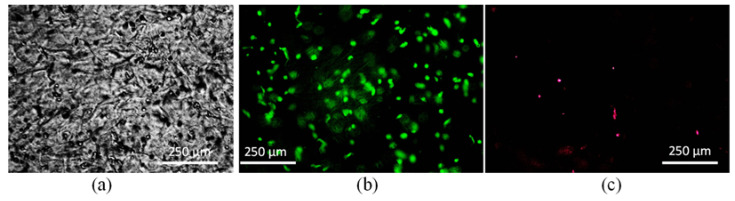
(**a**) Cell encapsulation with A_2_C_2_T_1_. Determining cell viability of bioink before printing (**b**) Live cell and (**c**) dead cell. It shows almost 95% cell viability before printing.

**Figure 18:**
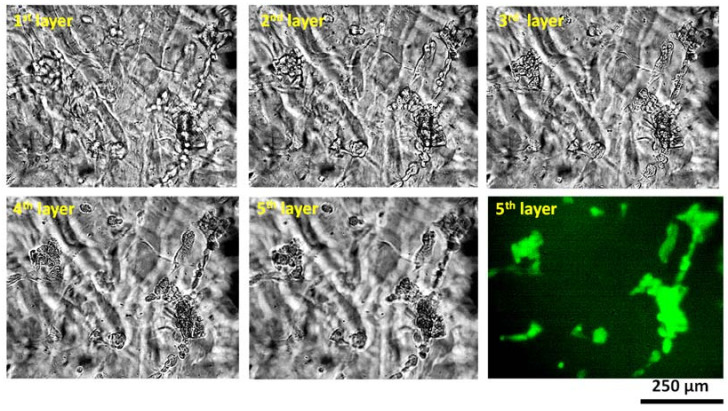
Height-based images captured to analyze the cell morphology. It was observed that cells started regaining their morphology after five incubation days.

As a representative of all compositions, we mentioned in this paper, we used A_2_C_2_TN_1_ to investigate its biocompatibility. Porc 1 cells are mixed uniformly with A_2_C_2_TN_1_, using a magnetic stirrer immediately prior to dispensing the hydrogel to fabricate a single filament. Before the printing of the bioink, the cell distribution and shape were observed under a fluorescent microscope (control). Most of the cells were round, and the cell distribution was almost uniform, as shown in Figure 17. It shows almost 95% cell viability before printing. Single filaments fabricated applying 8 psi air pressure using our prepared bioink showed 86% cell viability after five incubation days. Images were also captured at various heights into the filament to investigate the cell morphology as shown in Figure 18. It was observed that cells started regaining their morphology after five incubation days. This result supports our previously published analysis where we observed the the cell viability increased up to 90%, 89%, and 81%, respectively, for filaments fabricated with 8, 10, and 12 psi air pressures after 10 incubation days [22].

As an effort to explore the capacity to control the rheological properties of short-fiber TO-NFC with alginate and CMC, we prepared a large sample size of 46 compositions changing the percentage of TO-NFC from 0.005% to 1.0%, alginate from 1% to 4%, and CMC from 1% to 4%. We approximated the values of *n* and *K* using the Power-Law from the flow diagrams of those compositions that confirmed the shear thinning behavior of all compositions. The yield stresses and corresponding shear strains of each composition were determined using the Herschel–Bulkley equation where we clearly indicated the impact of various percentages of TO-NFC. This information is critical to select any bioink to 3D bioprint [46]. The amplitude and three-point thixotropic tests revealed the impact of TO-NFC on the LVR and recovery rate of the compositions. Moreover, the filament shape fidelity was analyzed focusing the impact of the solid content and print speed. SEM images revealed a uniform distribution of encapsulated TO-NFC fibers. In addition to that, alginate, CMC, and TO-NFC were distributed homogeneously and resulted in smooth cell structure. Finally, a biocompatibility test was conducted on A_2_C_2_T_1_ before and after the 3D bioprinting. Cell viability and morphology tests clearly indicated that alginate, CMC, and TO-NFC compositions are a safe house for live cells. This TO-NFC-based bioink can be a good addition to the 3D bioprintable bioink library. In the future, we will explore the impact of TO-NFC on other commonly used biomaterials for 3D bioprinting processes such as Gelatin, GelMA, Chitosan, Collagen, and Hyaluronic acid in terms of rheological and biological behavior. In addition to that, we plan to encapsulate various cell types into compositions other than A_2_C_2_TN_1_ to investigate their biocompatibility.

## 5. Conclusions

In this research, rheological analysis was conducted for a total of 46 compositions to identify the effect of various percentages of TO-NFC on the varying percentages of alginate and CMC. The flow diagrams were closely analyzed to determine shear thinning factors *n* and *K*. The impact of low and high concentrations of TO-NFC on *n* and *K* was demonstrated. The width of the fabricated filaments was analyzed and corelated with the values of *n*, *K*, and yield stress. To fabricate a large-scale functional tissue scaffold with the appropriate hybrid hydrogel, this experimental analysis can help identify correct materials to ensure geometrical fidelity by controlling the filament width. In the future, we will print filaments with all compositions, determine the filament width, and explore an analytical model enabling a relation between filament width and percentage of TO-NFC. The illustrated characterization techniques can direct the 3D bio-fabrication of the tailored anisotropic scaffolds, which will assist in future functional tissue fabrication.

## Figures and Tables

**Table 1:** Various compositions prepared with different weight percentages of alginate (1, 2, 3, and 4%, *w*/*v*), CMC (1, 2, 3, and 4%, *w*/*v*), and TO-NFC (0.005% and 0.5%, *w*/*v*).

(a)			
Alginate (A)	CMC (C)	TO-NFC (T)	Symbol
1	1	0.005	A_1_C_1_T_0.005_
	2		A_1_C_2_T_0.005_
	3		A_1_C_3_T_0.005_
	4		A_1_C_4_T_0.005_
2	1		A_2_C_1_T_0.005_
	2		A_2_C_2_T_0.005_
	3		A_2_C_3_T_0.005_
	4		A_2_C_4_T_0.5_
3	1		A_3_C_1_T_0.005_
	2		A_3_C_2_T_0.005_
	3		A_3_C_3_T_0.005_
4	1		A_4_C_1_T_0.005_
	2		A_4_C_2_T_0.005_
(b)			
**Alginate (A)**	**CMC (C)**	**TO-NFC (T)**	**Symbol**
1	1	0.5	A_1_C_1_T_0.5_
	2		A_1_C_2_T_0.5_
	3		A_1_C_3_T_0.5_
	4		A_1_C_4_T_0.5_
2	1		A_2_C_1_T_0.5_
	2		A_2_C_2_T_0.5_
	3		A_2_C_3_T_0.5_
	4		A_2_C_4_T_0.5_
3	1		A_3_C_1_T_0.5_
	2		A_3_C_2_T_0.5_
	3		A_3_C_3_T_0.5_
4	1		A_4_C_1_T_0.5_
	2		A_4_C_2_T_0.5_

**Table 2:** Various compositions prepared with different weight percentages of alginate (1, 2, 3, and 4%, *w*/*v*), CMC (1, 2, 3, and 4%, *w*/*v*), and TO-NFC (0.01% and 1.0%, *w*/*v*).

(a)			
Alginate (A)	CMC (C)	TO-NFC (T)	Symbol
1	1	0.01	A_1_C_1_T_0.01_
	2		A_1_C_2_T_0.01_
	3		A_1_C_3_T_0.01_
	4		A_1_C_4_T_0.01_
2	1		A_2_C_1_T_0.01_
	2		A_2_C_2_T_0.01_
	3		A_2_C_3_T_0.01_
3	1		A_3_C_1_T_0.01_
	2		A_3_C_2_T_0.01_
4	1		A_4_C_1_T_0.01_
(b)			
**Alginate (A)**	**CMC (C)**	**TO-NFC (T)**	**Symbol**
1	1	1	A_1_C_1_T_1_
	2		A_1_C_2_T_1_
	3		A_1_C_3_T_1_
	4		A_1_C_4_T_1_
2	1		A_2_C_1_T_1_
	2		A_2_C_2_T_1_
	3		A_2_C_3_T_1_
3	1		A_3_C_1_T_1_
	2		A_3_C_2_T_1_
4	1		A_4_C_1_T_1_

**Table 3:** Yield stress ($\tau_{0}$) and corresponding shear rate ($\dot{\gamma }$) prepared with different weight percentages of alginate (1, 2, 3, and 4%, *w*/*v*), CMC (1, 2, 3, and 4%, *w*/*v*), and TO-NFC (0.005%, 0.01%, 0.5%, and 1.0% *w*/*v*).

Symbol	SR	YS (Pa)	Symbol	SR	YS (Pa)	Symbol	SR	YS (Pa)	Symbol	SR	YS (Pa)
A_1_C_1_T_0.005_	0.199	0.28	A_1_C_1_T_0.5_	0.141	0.43	A_1_C_1_T_0.01_	0.141	0.35	A_1_C_1_T_1_	0.199	5.64
A_1_C_2_T_0.005_	0.282	2.65	A_1_C_2_T_0.5_	0.199	3.38	A_1_C_2_T_0.01_	0.282	4.47	A_1_C_2_T_1_	1.58	77.29
A_1_C_3_T_0.005_	0.794	31.33	A_1_C_3_T_0.5_	0.282	50.70	A_1_C_3_T_0.01_	0.398	16.64	A_1_C_3_T_1_	2.24	186.36
A_2_C_4_T_0.005_	3.16	189.40	A_1_C_4_T_0.5_	2.24	341.76	A_1_C_4_T_0.01_	0.562	69.61	A_1_C_4_T_1_	6.31	476.16
A_2_C_1_T_0.005_	0.398	3.78	A_2_C_1_T_0.5_	0.199	10.79	A_2_C_1_T_0.01_	0.141	1.585	A_2_C_1_T_1_	0.282	20.05
A_2_C_2_T_0.005_	0.794	22.32	A_2_C_2_T_0.5_	0.282	21.61	A_2_C_2_T_0.01_	0.199	7.92	A_2_C_2_T_1_	3.16	266.19
A_2_C_3_T_0.005_	3.16	141.22	A_2_C_3_T_0.5_	0.562	103.65	A_2_C_3_T_0.01_	0.282	24.34	A_2_C_3_T_1_	17.8	508.53
A_2_C_4_T_0.5_	8.91	403.84	A_2_C_4_T_0.5_	6.31	400.69	A_3_C_1_T_0.01_	0.282	15.97	A_3_C_1_T_1_	1.58	130.4
A_3_C_1_T_0.005_	0.794	22.71	A_3_C_1_T_0.5_	0.282	33.594	A_3_C_2_T_0.01_	0.794	46.80	A_3_C_2_T_1_	2.24	302.48
A_3_C_2_T_0.005_	1.12	100.42	A_3_C_3_T_0.5_	1.12	111.49	A_4_C_1_T_0.01_	1.58	146.09	A_4_C_1_T_1_	6.31	799.96
A_3_C_3_T_0.005_	8.91	340.51	A_3_C_3_T_0.5_	3.16	304.04						
A_4_C_1_T_0.005_	3.16	150.75	A_4_C_1_T_0.5_	1.12	226.71						
A_4_C_2_T_0.005_	12.6	412.70	A_4_C_2_T_0.5_	17.8	540.36						

## Data Availability

Not applicable.
